# An Oblique Columellar Artery Variant

**DOI:** 10.7759/cureus.1958

**Published:** 2017-12-18

**Authors:** Paul J Choi, Joe Iwanaga, R. Shane Tubbs

**Affiliations:** 1 Clinical Anatomy, Seattle Science Foundation; 2 Seattle Science Foundation; 3 Neurosurgery, Seattle Science Foundation

**Keywords:** anatomy, cadaver, rhinoplasty, reconstruction, nasal septum

## Abstract

A variant of the columellar artery (CoA), also known as the nasal septal branch of the superior labial artery of the facial artery, and its unusual direct anastomosis with the right infraorbital artery were identified in a cadaveric dissection. Numerous variants and distribution patterns of the CoA have been described in the literature. However, an oblique and a third CoA variant that anastomoses with the contralateral infraorbital artery has never been reported or depicted. We highlight the significance of a preprocedural vascular examination of the perioral region for optimal functional and esthetic outcome by presenting this unique case. This CoA variant should be known by surgeons performing invasive surgical techniques in the columellar region.

## Introduction

The septal nasal cartilage (SC) and the tip of the nose receive their arterial supply from the branches of the external and internal carotid arteries. The external carotid artery gives rise to the maxillary and facial arteries, which anastomose at the nasal septal branches of the sphenopalatine artery and the superior labial artery, respectively [1]. The superior labial artery, a branch of the facial artery arising at the level of the labial commissure, is the main blood supply to the upper lip and provides a septal branch, the columellar arteries (CoAs) [2-4]. Thus, the upper labial region has a rich and variable blood supply [2], and anatomical knowledge of the course of the CoAs and its variation is required in order to complete better perioral surgery, such as microsurgical repair of the lips and rhinoplasty [2,5]. We report a case of the variant course of the additional CoA, which directly anastomosed with the contralateral infraorbital artery.

## Case presentation

**Figure 1:**
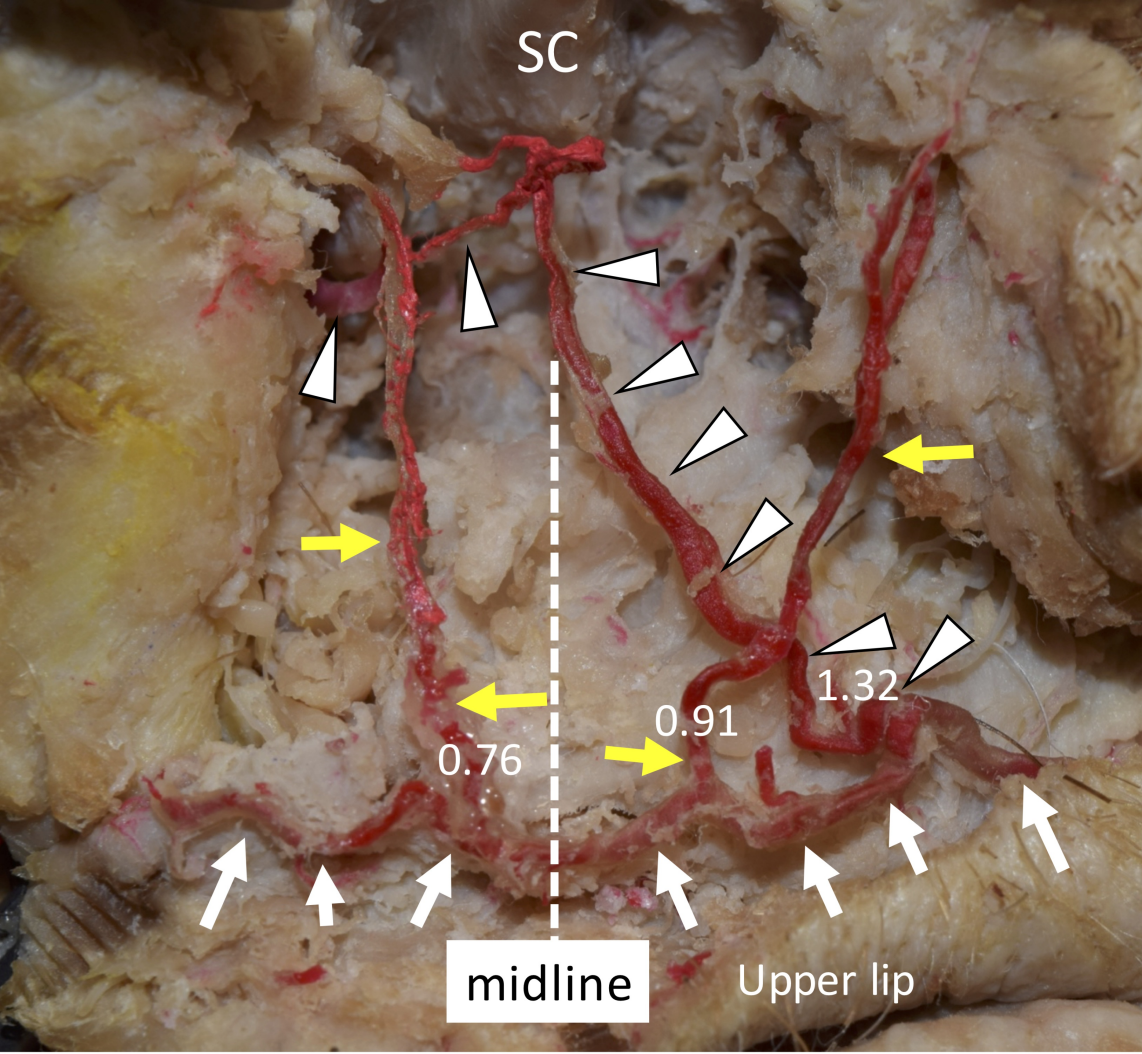
A variant of the nasal septal branch of the superior labial artery A variant of the nasal septal branch of the superior labial artery (ala is reflected laterally). The right and left superior labial arteries (white arrows) connect and give rise to the right and left nasal septal branches (yellow arrows). Note that another large branch (arrowheads) arises from the left superior labial artery, runs mediosuperiorly, and crosses the midline. SC; septal nasal cartilage

**Figure 2:**
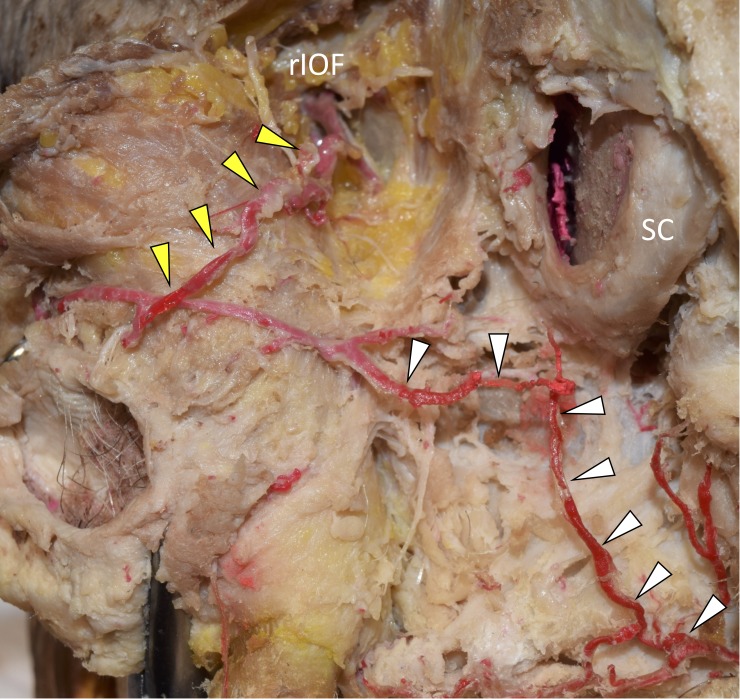
The variant nasal septal branch anastomoses with the right infraorbital artery The variant nasal septal branch (white arrowheads) anastomoses with the infraorbital artery (yellow arrowheads). rIOF; right infraorbital foramen, SC; septal nasal cartilage

We performed a superficial dissection of the face of a 73-year-old at death Caucasian male, a fresh frozen cadaver, and performed anatomical measurements with a microcaliper (Mitutoyo, Kanagawa, Japan). At the angle of the left mandible, the left facial artery was located and carefully dissected to follow its branches, i.e., the superior labial artery and the CoAs. An unusual third CoA, which ascended obliquely from the columellolabial junction toward the right of the SC, i.e., across the midline mediosuperiorly (Figure 1), was located in the philtrum along with two other CoAs that symmetrically ascended in a vertical fashion (the columellolabial junction of the right vertical CoA was 0.76 mm in diameter and that of the left vertical CoA was 0.91 mm in diameter, respectively), following the pattern of a common columellar variation [5]. The columellolabial junction of the oblique variant was more distal from the SC than that of the left vertical CoA, i.e., 1.32 mm in diameter (Figure 1). Further dissection of the oblique CoA revealed an unusual direct aastomosis with the infraorbital artery (Figure 2).

## Discussion

The sphenopalatine artery, a branch of the maxillary artery, provides the major blood supply to the SC and its overlying mucosa [6-7]. This artery gives rise to the medial septal artery, which exits the sphenopalatine foramen located at the insertion site of the middle concha and travels anteroinferiorly toward the wall of the sphenoidal sinus to ultimately reach the inferior septal area [6-8]. The sphenopalatine artery then contributes three branches to the septal circulation and anastomoses with the facial artery via the superior labial artery [8].

The internal carotid artery also contributes to the vascular system of the nasal septum via its ophthalmic branch, which joins the infraorbital artery as it descends to the septum [1,5]. However, to our knowledge, the direct anastomosis between the infraorbital artery and the CoA, especially the contralateral CoA, as seen in the present case, has not been reported.

The course, shape, quantity, and anastomotic nature of the septal arterial supply are highly variable and, therefore, a profound understanding of the anatomy is crucial in the management of maxillofacial anomalies of the perioral region, an area that is also susceptible to trauma and carcinoma, and an intractable epistaxis with minimal surgical failure [2-5]. For instance, Magden et al. reported that 29% of superior labial arteries originate from the facial artery unilaterally [2]. They also reported that 36% of the studied cadavers did not have any septal branches [2]. Further, the CoA is often transected during external rhinoplasty [5,9-10]. However, due to the preservation of the lateral nasal artery working as the primary supply to the nasal tip with variable and less significant contribution from the CoA(s) and possible collateral branches from the ophthalmic artery, ischemia of the tip usually does not occur [5,10].

## Conclusions

The arterial distribution of the columellar region of the perioral area is highly variable. A third, oblique CoA variant, which directly anastomoses with the contralateral infraorbital artery, has never been described in the literature until now. Recognition and an understanding of this unusual arterial pattern are important in safely performing surgical procedures in this area of the face.
